# Assessing the clinical diagnostic utility of multiplex ddPCR assays in thyroid nodules

**DOI:** 10.1186/s13044-026-00292-9

**Published:** 2026-03-20

**Authors:** Xiubo Li, Chunhui Shen, Xiang Peng, Qianfeng Xu, Jun Zhang, Minghui He, Minyi Kong, Zhao Lin, Jingyan Luo, Yan Wang

**Affiliations:** 1 Department of Pathology, Guangzhou First People’s Hospital, School of Medicine, South China University of Technology, Guangzhou, China; 2 Center for Medical Research on Innovation and Translation, Institute of Clinical Medicine, Guangzhou First People’s Hospital, School of Medicine, South China University of Technology, Guangzhou, China; 3 Department of Thoracic and Cardiovascular Surgical, The First Affiliated Hospital of Guangzhou University of Chinese Medicine, Guangzhou, China; 4 Department of Pathology, Guangdong Provincial People’s Hospital, Zhuhai Hospital (Jinwan Central Hospital of Zhuhai), Zhuhai, China; 5 Department of Urology, Guangzhou First People’s Hospital, The Second Affiliated Hospital, School of Medicine, South China University of Technology, Guangzhou, China; 6 Department of Liver, Gallbladder and Pancreatic Surgery, The Second Affiliated Hospital, School of Medicine, South China University of Technology, Guangzhou, China; 7 Forevergen Biosciences Center, Guangzhou, China; 8 Guangdong Forevergen Medical Technology Co., Ltd., Foshan, China

## Abstract

**Background:**

Ultrasound-guided fine-needle aspiration cytology (FNAC) is the standard method for evaluating thyroid nodules, but 20–40% of specimens are still cytologically indeterminate (Bethesda III–IV), leading to repeat biopsies or diagnostic lobectomy. We hypothesized that a multiplex droplet digital PCR (ddPCR) assay covering key thyroid-cancer driver mutations would increase the pre-operative diagnostic yield.

**Methods:**

A six-gene ddPCR panel was developed to detect *BRAF* p.V600E, *TERT* promoter mutations c.-124 C > T (C228T) and c.-146 C > T (C250T), *PIK3CA* p.H1047R/L, and all clinically relevant variants in *NRAS*, *HRAS*, and *KRAS* at codons 12, 13, and 61. Analytical performance was established using plasmid controls. The assays were subsequently applied to 210 prospectively collected fine-needle aspiration (FNA) specimens from 201 patients, with parallel ARMS-PCR testing for *BRAF* p.V600E and histopathological confirmation available for 49 surgically excised nodules.

**Results:**

The six-gene ddPCR panel demonstrated 100% analytical specificity and a limit of detection of 0.1% variant allele frequency (VAF). In clinical samples, 103 of 210 nodules (49%) harbored at least one mutation, with *BRAF* p.V600E being the most prevalent alteration (81/103, 78.6%). ddPCR identified five low-abundance *BRAF* p.V600E mutations (VAF 0.37–2.96%) that were missed by ARMS-PCR, one of which was confirmed as papillary thyroid carcinoma on postoperative pathology. Mutation prevalence increased progressively across Bethesda categories, ranging from 33% in Bethesda I to 89% in Bethesda VI. Compared with surgical pathology, the combination of the six-gene ddPCR panel and FNAC achieved 100% sensitivity, 71% specificity, and 96% diagnostic accuracy, significantly outperforming FNAC alone or FNAC combined with ARMS-PCR (*P* < 0.05).

**Conclusions:**

This study developed a six-gene mutation detection panel composed of four multiplex droplet digital PCR (ddPCR) assays for thyroid nodule analysis. Integration with cytology substantially reduces indeterminate diagnoses and may guide personalized management, including the avoidance of unnecessary surgery.

**Supplementary Information:**

The online version contains supplementary material available at 10.1186/s13044-026-00292-9.

## Introduction

Thyroid tumors rank among the most prevalent tumors within the endocrine system [1, 2]. Recently, as health awareness has increased, the detection rate of thyroid nodules during health check-ups ranges from 20% to 76% by ultrasound techniques, but only 7% to 15% of these nodules are malignant [2, 3]. Fine-needle aspiration cytology (FNAC) under ultrasound guidance is an important method for assessing the benignity or malignancy of thyroid nodules. Performing FNAC before surgery can effectively increase the proportion of malignant tumors in thyroid surgeries and avoid unnecessary operations [4]. However, FNAC is influenced by factors such as sampling site, nodule size, and the experience of pathologists, resulting in randomness and a certain degree of false-negative rate. Additionally, about 20% to 40% of nodules yield indeterminate results (Bethesda categories III–IV), leading to repeated biopsies or diagnostic lobectomy [4, 5].

Increasing research indicates that Integrating FNAC with molecular testing enhances the diagnostic accuracy of thyroid nodules [5–7]. Currently, the main platforms used in clinical settings for detecting gene variations in thyroid cancer are real-time fluorescent quantitative PCR (qPCR) and next-generation sequencing (NGS) [8, 9]. qPCR is simple to operate, provides quick results, and has relatively high sensitivity, making it commonly used for detecting mutations like *BRAF* p.V600E [10],7-gene panel [11] and 5-gene panel [12]. However, the sensitivity of ARMS-PCR was frequently insufficient, ranged from 61% to 69%, owing to the generally low neoplastic cellularity of FNA specimens [11–13]. NGS, which commercial molecular testing panels for indeterminate thyroid nodules include Thyroseq [14], ThyGeNEXT/ThyraMIR [15], and Afirma GSC, offers high throughput and a broad detection range but is limited by high cost, long turnaround time, and highly specialized laboratories [16]. Digital PCR (dPCR), an absolute quantitative technique based on massive micro-partitioning and Poisson statistics, offers markedly Current ddPCR assays typically target single genes (e.g., *BRAF*), missing the clinical impact of multiplex panels that include *RAS*,* TERT* and *PIK3CA*—mutations present in > 30% of indeterminate nodules linked to aggressive disease [17–20].Increasing evidence indicates that co-occurring genetic alterations, rather than individual mutations in isolation, confer a higher risk of aggressive disease phenotypes and unfavorable clinical outcomes [21–23].When surveying mutation-rich hotspots, the limited fluorophore palette of ddPCR becomes restrictive. Drop-off assays overcome this by using two wild-type-specific probes: dual binding gives a strong double-positive signal, whereas any mismatch prevents one probe from annealing, producing a “drop-off” signal that accurately reveals the variant [24, 25]. In the present study, we developed and validated a multiplex drop-off ddPCR panel targeting *BRAF* p.V600E, *PIK3CA* p.H1047R/L, *TERT* promoter mutations c.-124 C > T (C228T) and c.-146 C > T (C250T), as well as all clinically relevant alterations in *KRAS*, *NRAS*, and *HRAS* at codons 12 (G12), 13 (G13), and 61 (Q61). By integrating FNAC, multi-gene ddPCR analysis, and postoperative histopathology, we aimed to evaluate the clinical utility of this multiplex assay for preoperative discrimination between benign and malignant thyroid nodules.

## Materials and methods

### Patients and samples

A total of 201 patients, presenting with 210 thyroid nodules, who underwent ultrasound-guided fine-needle aspiration cytology (FNAC) and *BRAF* p.V600E testing using ARMS-PCR at the Guangzhou First People’s Hospital from January 2022 to June 2023, were retrospectively included in this study. The cohort comprised 64 males and 137 females, with a mean age of 46.16 ± 13.54 years. Inclusion criteria were as follows: [1] patients who consented to preoperative thyroid ultrasound, FNAC, and genetic testing; [2] patients possessing complete pathological description data. Exclusion criteria included: [1] patients with a history of thyroid dysfunction who were receiving pharmacological treatment (e.g., anticoagulant therapy) or whose thyroid function was unstable; [2] patients with a history of thyroid surgery or documented distant metastasis; [3] patients with coagulation abnormalities, which represent relative contraindications for fine-needle aspiration procedures rather than factors that directly affect molecular testing results; [4] patients with incomplete lesion data or who were unable to cooperate during FNAC. Exclusions were therefore based primarily on clinical safety considerations during FNA sampling. The study was conducted in accordance with the principles of the Declaration of Helsinki. Written informed consent was obtained from all participants, and the study protocol was approved by the Medical Ethics Committee of Guangzhou First People’s Hospital (Approval No. K-2025-034-01).

### Fine needle aspirations (FNAs)

Patients were positioned supine with a pillow under the shoulders and the head slightly tilted back to expose the neck. The puncture site was disinfected using standard aseptic techniques. Given the fine gauge of the needles (22–25G), local anesthesia was generally not applied, though topical anesthetic could be used for anxious or sensitive patients.

Under real-time ultrasound guidance, the needle was precisely inserted into the target nodule, avoiding large vessels and critical structures. Suspicious regions, such as microcalcifications or solid areas, were preferentially sampled. Sampling was performed using either the aspiration (negative pressure) or capillary (non-aspiration) technique. Each nodule was sampled 2–4 times from different areas, with rapid small-amplitude (2–3 mm) needle movements to optimize cellular yield. During each aspiration, the collected material was simultaneously divided, with one portion submitted for routine cytological evaluation and a small aliquot reserved for molecular analysis. No additional or dedicated FNA passes were performed specifically for genetic testing.

The samples designated for molecular analysis were immediately transferred into a cell preservation solution provided with the DNA extraction kit (AmoyDx, Xiamen AmoyDx Diagnostics Co., Ltd., Xiamen, China) and processed according to the manufacturer’s instructions. This commercially available preservation reagent is validated for stabilizing nucleic acids and ensuring compatibility with downstream molecular assays, including ddPCR and gene sequencing.

### Fine-needle aspiration cytology (FNAC)

FNAC results were classified according to *The Bethesda System for Reporting Thyroid Cytopathology (TBSRTC)* into six diagnostic categories: [1] Category I, Non-diagnostic (ND) or Unsatisfactory (UNS); [2] Category II, Benign lesion; [3] Category III, Atypia of Undetermined Significance (AUS) or Follicular Lesion of Undetermined Significance (FLUS); [4] Category IV, Follicular Neoplasm (FN) or Suspicious for a Follicular Neoplasm (SFN); [5] Category V, Suspicious for Malignancy; [6] Category VI, Malignant.

### Nucleic acid extraction

The Tissue DNA Extration Kit (Xiamen AmoyDx Co., Ltd., China) was utilized to extract DNA from thyroid nodule FNAC samples. A UV spectrophotometer was used to evaluate the concentration and quality of the extracted DNA, ensuring the A260/A280 absorbance ratio fell between 1.8 and 2.0, and the DNA was stored at -20 °C for later use.

### Detection of *BRAF* p.V600E mutation by ARMS-PCR

The human *BRAF* p.V600E mutation detection kit (Xiamen AmoyDx Co., Ltd., China) was utilized to detect the *BRAF* p.V600E mutation. This kit has a sensitivity of 1% for gene mutation detection, as stated in the manufacturer’s instructions. The *BRAF* p.V600E mutation was identified in accordance with the manufacturer’s recommended protocol.

### Six-gene detection by digital droplet PCR (ddPCR)

All primers and probes of the Six-Gene ddPCR assays (Table 1) were designed using Primer Express 3.0.1 software (Thermo Fisher Scientific). The Six-Gene ddPCR assays were employed to detect the *BRAF* p.V600E mutation, the *PIK3CA* p.H1047R and p.H1047L mutations, *TERT* promoter mutations (C228T and C250T), as well as hotspot mutations in the *NRAS*, *HRAS*, and *KRAS* genes at codons 12, 13, and 61. Droplet digital PCR was performed using the MicroDrop-400™ Digital Droplet PCR System (Forevergen, China), according to the manufacturer’s instructions. The reaction mixture included 5 µL of 4×SuperMix for Probe (Forevergen, China), 2 µL of a 10×primer and probe mixture, 3 µL of a PCR enhancer (1mM Na2EDTA and 0.5mM betaine), 6 µL of ddH2O, and 4 µL of template DNA.The DNA input for each ddPCR reaction was 20 ng per reaction. Thermal cycling conditions involved 10 min at 95 ℃; 45 cycles of 30 s at 95 ℃ and 60 s at 60 ℃; and a final cycle of 98 ℃ for 10 min, ending with a hold at 16 ℃. The lower limits of detection (LOD) and quantification (LOQ) were established at 0.1% variant allele frequency. A sample was considered positive when three or more mutation-positive droplets were detected.

**Table 1:** Probes and primers used for the six-gene ddPCR assays

Oligo name	No	Sequence(5’-3’)
*PIK3CA* p.H1047R-F	SEQ ID No.1	TAAAACTGAGCAAGAGGCTTTGGAGTA
*PIK3CA* p.H1047R-R	SEQ ID No.2	AATTGTGTGGAAGATCCAATCC
*BRAF* p.V600E-F	SEQ ID No.3	GAGATCTACTGTTTTCCTTTACTTACTACA
*BRAF* p.V600E-R	SEQ ID No.4	GGATCCAGACAACTGTTCAAACTGATGG
*TERT* F1	SEQ ID No.5	TCCAGCTCCGCCTCCTCCGCGC
*TERT* R5	SEQ ID No.6	TGAAACTCGCGCCGCGAGG
dd-*RPP30*-F	SEQ ID No.7	GCGGTGTTTGCAGATTTGGAC
dd-*RPP30*-R	SEQ ID No.8	ACTCACGGTGAGCGGCTGTCTCCA
*NRAS* exon3-F	SEQ ID No.9	CTTGCTATTATTGATGGCAAATACACA
*NRAS* exon3- R	SEQ ID No.10	CAAGTGGTTATAGATGGTGAAACCTGTTT
*NRAS* exon2-F	SEQ ID No.11	ACCTCTATGGTGGGATCATATTCATC
*NRAS* exon2-R	SEQ ID No.12	ATGACTGAGTACAAACTGGTG
*HRAS* exon3-F	SEQ ID No.13	CCGGAAGCAGGTGGTCAT
*HRAS* exon3-R	SEQ ID No.14	AGGAAGCCCTCCCCGGT
*HRAS* exon2-F	SEQ ID No.15	GGAGCGATGACGGAATATAAGC
*HRAS* exon2-R	SEQ ID No.16	GGGGTCGTATTCGTCCACAA
*KRAS* exon3-F	SEQ ID No.17	GATGGAGAAACCTGTCTCTTGGA
*KRAS* exon3-R	SEQ ID No.18	ATACACAAAGAAAGCCCTCCCCAG
*KRAS* exon2-F	SEQ ID No.19	TAAGGCCTGCTGAAAATGACTGAATATAA
*KRAS* exon2-R	SEQ ID No.20	TATTGTTGGATCATATTCGTCCACAAA
*PIK3CA* p.H1047R-MT	SEQ ID No.21	FAM-AACAAATGAATGATGCACGTCAT-MGB
*PIK3CA* p.H1047L-MT	SEQ ID No.22	FAM-AATGATGCACTTCATGGTGGCTG-MGB
*PIK3CA* p.H1047 Blocker	SEQ ID No.23	TGATGCACATCATGGTGGCTGGA-MGB
*BRAF* p.V600E	SEQ ID No.24	VIC-TCCATCGAGATTTCTCTGTAGCTAGAC-BHQ1
*TERT*(C228T) Probe	SEQ ID No.25	CY5-CCAGCCCCTTCCGGGCCCTCCCAG-BHQ3
*TERT*(C250T) Probe	SEQ ID No.26	SX670-GCCCCGTCCCGACCCCTTCCG-BHQ3
*TERT* C228 Blocker	SEQ ID No.27	CAGCCCCCTCCGGGCCCTC-ddC
*TERT* C250 Blocker	SEQ ID No.28	GTCCCGACCCCTCCCGGGTCCC-ddC
dd-*RPP30*-Probe	SEQ ID No.29	CY5.5-CGGGTTCTGACCTGAAGGCTCTGC-BHQ3
*NRAS*-exon3-wt-P	SEQ ID No.30	FAM-ACAGCTGGACAAGAAGAGTACAGT-MGB
*NRAS*-exon3-ref-P	SEQ ID No.31	VIC-GAGACCAATACATGAGGACAGG-MGB
*NRAS*-exon2-wt-P	SEQ ID No.32	CY5-AGCAGGTGGTGTTGGGAAAAGCGC-BHQ3
*NRAS*-exon2-ref-P	SEQ ID No.33	CY5.5-TGACAATCCAGCTAATCCAGAACCA-MGB
*HRAS* exon3-wt-P	SEQ ID No.34	FAM-CGCCGGCCAGGAGGAGTACA-MGB
*HRAS* exon3-ref-P	SEQ ID No.35	VIC-GCCTGTTGGACATCCTGGA-MGB
*HRAS* exon2-wt-P	SEQ ID No.36	CY5.5-CGGCGGTGTGGGCAAGAGTG-BHQ3
*HRAS* exon2-ref-P	SEQ ID No.37	CY5-TGACCATCCAGCTGATCCAGAACC-BHQ3
*KRAS* exon3-wt-P	SEQ ID No.38	VIC-CTCCTCTTGACCTGCTGTGTCGA-MGB
*KRAS* exon3-ref-P	SEQ ID No.39	FAM-TCATGTACTGGTCCCTCATTGC-MGB
*KRAS* exon2-ref-P	SEQ ID No.40	CY5-AGTGCCTTGACGATACAGCTAAT-BHQ3
*KRAS* exon2-wt-P	SEQ ID No.41	CY5.5-AGTTGGAGCTGGTGGCGTAGG-BHQ3

*FAM‌: Fluorescein Amide; ‌VIC‌ VIC-Amidite; ‌CY5‌ Cyanine 5;‌CY5 5‌ Cyanine 5.5; wt-P, wild type probe; Ref-P, reference probe; BHQ, black hole quencher; MGB, minor groove binder; ddC: DiDeoxy Cytidine*

### Development of the six-gene ddPCR assays

**Fig. 1:**
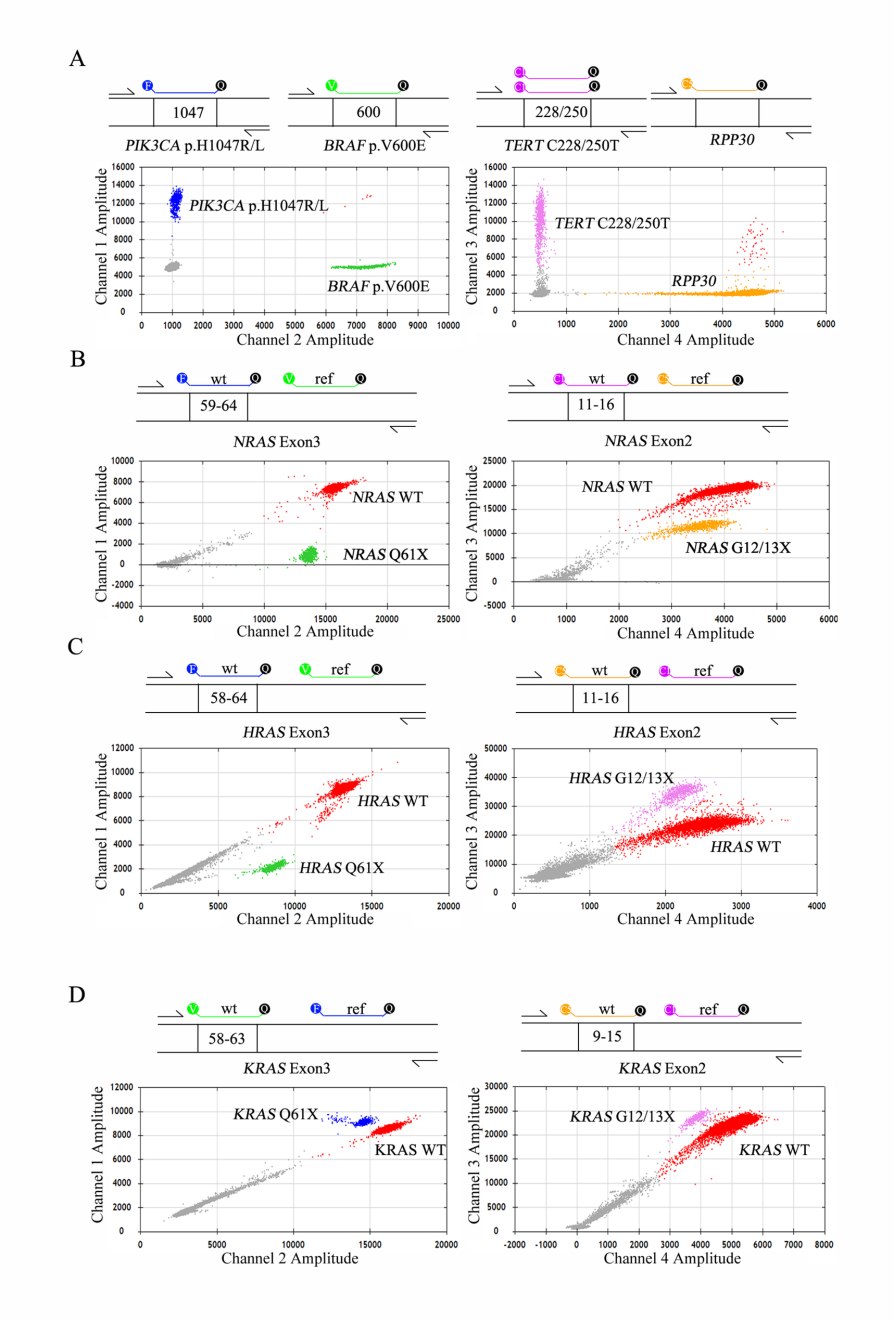
Design of the six-gene ddPCR assays**.** **A**. Schematic representation and two-dimensional (2D) dot plots for the assays aimed at identifying mutations in *BRAF* p.V600E, *PIK3CA* p.H1047R/L, and *TERT* C228/250T, with *RPP30* utilized as the reference gene. In this representative 2D figure, signals for *PIK3CA* p.H1047R/L mutants are indicated in Channel 1 (FAM), signals for *BRAF* p.V600E mutants appear in Channel 2 (VIC), Channel 3 captures signals for *TERT* C228/250T mutants (CY5), and Channel 4 shows signals for the *RPP30* gene (CY5.5). **B**-**D**. Schematic designs and 2D dot plots for the drop-off assays pertaining to *NRAS*, *HRAS* and *KRAS*. The drop-off principle: Wild-type (WT) sequences are identified by both a wt specific probe and a reference probe, resulting in a cluster of double-positive (red) droplets on the 2D dot plot. Conversely sequences containing mutations within the hotspot region can only be recognized by the reference probe

Four multiplex ddPCR assays were developed using a combination of conventional TaqMan chemistry and drop-off ddPCR methodologies. The *BRAF–PIK3CA–TERT* assay integrates three traditional TaqMan systems to detect *BRAF* p.V600E, *PIK3CA* p.H1047R/L, and *TERT* promoter mutations c.-124 C > T (C228T) and c.-146 C > T (C250T), using *RPP30* as the internal reference (Fig. 1A). The *NRAS* assay comprises two drop-off systems (Fig. 1B): one targeting the wild-type nucleotide sequence spanning codons 59–64 using a FAM-labeled drop-off probe paired with a VIC-labeled reference probe, and another targeting codons 11–16 using CY5 and CY5.5 probes within the same amplicon. Similarly, the *HRAS* assay includes two drop-off systems covering codons 58–64 and codons 11–16 (Fig. 1C). The *KRAS* assay targets sequence at codons 58–63 and codons 9–15 region(Fig. 1D). Based on the COSMIC database, these four ddPCR assays collectively cover over 98.32%, 98.55%, 16.39%, 85.24%, 95.37%, and 88.04% of all known pathogenic mutations in *BRAF*,* TERT*,* PIK3CA*,* NRAS*,* HRAS*, and *KRAS*, respectively, in thyroid tumors.

### Assay optimization and analytical performance

To improve amplification efficiency in high-GC regions such as the TERT promoter, the reaction mixture was supplemented with 1 mM Na₂EDTA and 0.5 mM betaine.

#### Limit of Blank (LOB) and Limit of Detection (LOD)

The LOB for each mutation was determined by analyzing 10 replicates of no-template controls (NTCs). All assessed mutations, including TERT, demonstrated an LOB of 0 copies/20 µL.Serial dilutions of mutant plasmids were prepared in a background of 15,000 copies/20 µL wild-type genomic DNA to evaluate assay sensitivity. Each dilution level (theoretical variant allele frequencies of 0.2%, 0.1%, and 0.05%) was tested in 10 replicates. Based on probit analysis, the 95% limit of detection (LOD) for all mutations was determined to be 0.1%.

#### Linearity and reproducibility

**Fig. 2:**
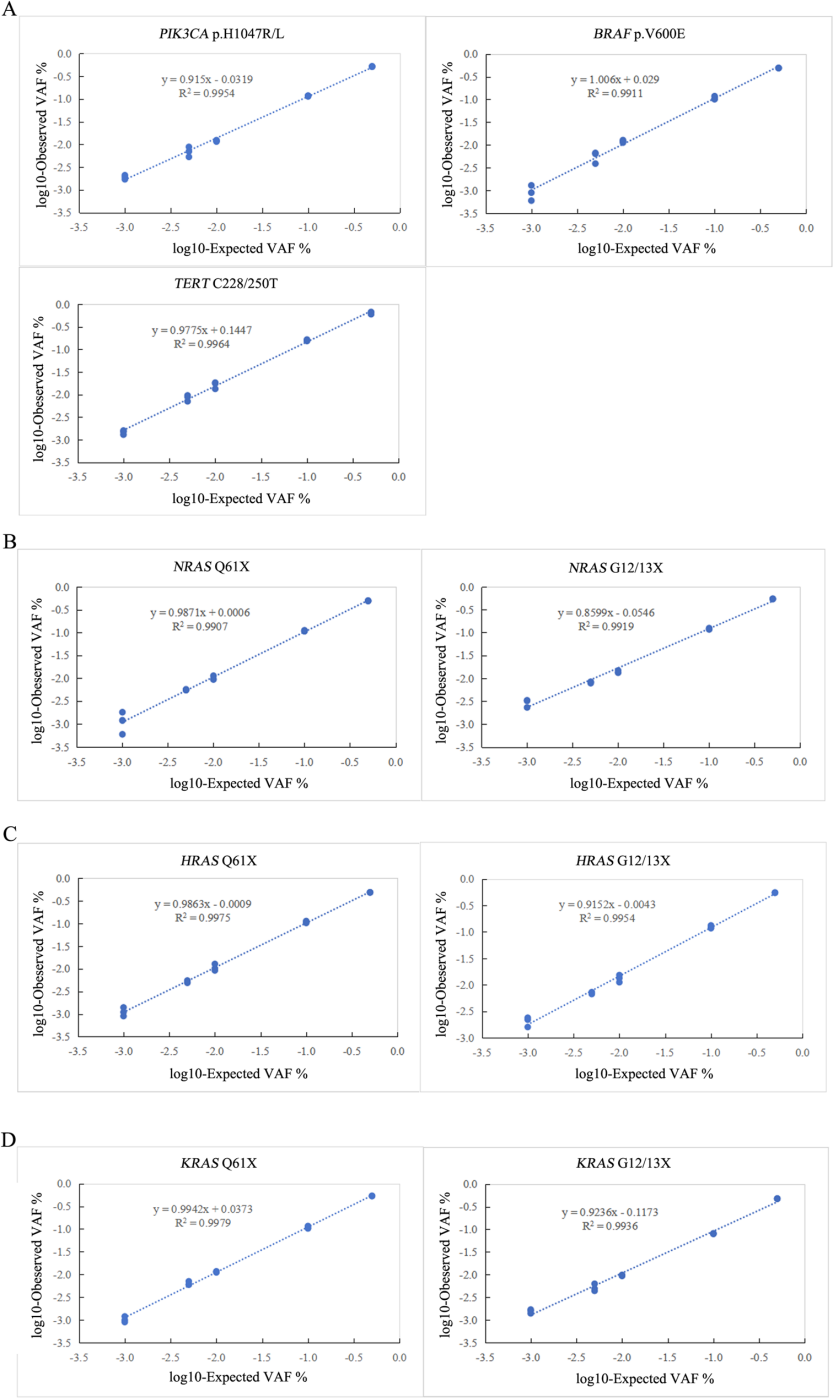
Presents the linearity analysis of the six-gene ddPCR assays**. A-D. **Each assay’s DNA mixes were prepared through serial dilutions of mutant plasmids representing each mutation classification within a constant wild-type genomic DNA backdrop of 15,000 copies/PCR aimed at achieving theoretical VAFs of 50%, 10%, 1%, 0.5% and 0.1%. Each DNA mix underwent triple testing. The Pearson correlation between observed and expected VAFs was computed using linear regression

Linearity was assessed across theoretical VAFs ranging from 50% to 0.1%, tested in triplicate. All mutations achieved correlation coefficients ≥ 0.99 between expected and observed VAFs (Fig. 2A–D). Reproducibility was confirmed across different VAF levels: VAF10%: 2.18%–10.59%; VAF1%: 2.76%–17.21%;VAF0.1%: 10.53%–58.53%.These data collectively demonstrate robust sensitivity, linearity, and reproducibility of the six-gene ddPCR assays.

### Statistical analysis

SPSS version 22 software (IBM, USA) was used for statistical analyses. Continuous variables that were normally distributed are presented as the mean ± standard deviation (‾*x* ± *s*) and were evaluated using the t-test. Categorical variables were presented as frequencies (n (%)) and the chi-square (χ2) test was employed for analysis. The effectiveness of the six-gene ddPCR assays in FNACs was evaluated by determining sensitivity, specificity, positive predictive value (PPV), negative predictive value (NPV), and accuracy. Statistically significant differences were identified by a p-value of less than 0.05.

## Results

### Analytical performance summary

The analytical performance of the six-gene ddPCR assays—including LOB, LOD, linearity, and reproducibility—was evaluated as described in the Methods section. All assays achieved a 95% LOD of 0.1%, demonstrated excellent linearity (R^2^≥ 0.99), and showed consistent reproducibility across varying VAF levels (Fig. 2).

### Clinical and cytological findings of FNA specimens

The subsequent phase involved testing the six-gene ddPCR assays on DNA extracted from thyroid nodule FNA samples. A total of 210 FNA nodules from 201 patients, all of whom had both FNA cytology and ARMS-PCR *BRAF* p.V600E testing available were included in this study. The average age of the participants was 46.16 ± 13.54 years, with 68.57% being female. According to the TBSRTC system, 55 cases (26.19%) were classified as malignant tumor (category VI), while 28 cases (13.33%) were deemed suspicious for malignancy (category V). Additionally, 46 cases (21.91%) fell into categories III/IV, presenting diagnostic challenges, and 78 cases (37.14%) were categorized as TBSRTC II, indicating benign nodules; 3 cases (1.43%) were categorized as nondiagnostic (category I). A detailed explanation of the Bethesda classification is presented in Table 2.

**Table 2:** Comparison of the clinical and molecular characteristics of participants with surgery versus those without

	Total (*n* = 210)	With surgery (*n* = 49)	Loss to surgical (*n* = 161)	*P* value
Age, years	46.16 ± 13.54	48.18 ± 13.04	45.82 ± 13.53	0.469
Sex				
Male	66(31.43%)	17(34.69%)	49(30.43%)	0.574
Female	144 (68.57%)	32(65.31%)	112(69.57%)	
TI-RADS grading				0.001
NA	68(32.38%)	11(22.45%)	57(35.40%)	
1 ~ 2	6 (2.86%)	2(4.08%)	4(2.48%)	
3	33(15.71%)	7(14.29%)	26(16.15%)	
4	94 (44.76%)	22(44.90%)	72(44.72%)	
5–6	9(4.28%)	7(14.29%)	2(1.24%)	
Nodules number				0.081
NA	68 (32.38%)	11(22.45%)	57(35.40%)	
1	44 (20.95%)	16(32.65%)	28(17.39%)	
2	25 (11.90%)	7(14.29%)	18(11.18%)	
≥ 3	73 (34.76%)	15(30.61%)	58(36.02%)	
FNA Bethesda classification				< 0.001
I: ND/UNS	3 (1.43%)	0	3(1.86%)	
II: benign	78 (37.14%)	2(4.08%)	76(47.20%)	
III: AUS/FLUS	43 (20.48%)	7(14.29%)	36(22.36%)	
IV: FN/SFN	3 (1.43%)	2(4.08%)	1 (0.62%)	
V: SM	28(13.33%)	12(24.49%)	16(9.94%)	
VI: maligancy	55 (26.19%)	26(53.06%)	29 (18.01%)	
ARMS-PCR-*BRAF* p.V600E				< 0.001
Positive	76(36.19%)	32(65.31%)	44(27.33%)	
Negative	134(63.81%)	17(34.69%)	117(72.67%)	
ddPCR-*BRAF* p.V600E				< 0.001
Positive	81(38.57%)	33(67.35%)	49(30.43%)	
Negative	129(61.43%)	16(32.65%)	112(69.57%)	
ddPCR -six genes				< 0.001
Positive	103(49.05%)	38(77.55%)	65(40.37%)	
Negative	107(50.95%)	11(22.45%)	96(59.63%)	

RADS: The Thyroid Imaging Reporting and Data System (TI-RADS) [26] classifies nodes as follows: 1 indicates a normal thyroid gland; 2 denotes benign conditions with a 0% risk of malignancy; 3 suggests probably benign nodules with less than a 5% malignancy risk; 4 refers to suspicious nodules with a malignancy risk ranging from 5% to 80%; 5 indicates probably malignant nodules with a greater than 80% risk; and 6 categorizes biopsy-proven malignancy

Bethesda classification [27]: I represents nondiagnostic/unsatisfactory specimens (ND/UNS); II signifies benign; III indicates atypia of undetermined significance or follicular lesion of undetermined significance (AUS/FLUS); IV refers to follicular neoplasm or suspicious for a follicular neoplasia (FN/SFN); V categorizes suspicious for malignancy (SM); and VI signifies malignancy. Abbreviations include FNA for fine-needle aspirate and NA for not available

Among the 210 nodules, 49 underwent surgical intervention, while 161 did not receive surgical treatment. The grading of TI-RADS and the FNA classification for the samples in the surgical group were significantly higher than those in the non-surgical group, with differences that were statistically significant (*P* ≤ 0.001). Furthermore, the rates of gene mutation detected in the surgical group using both ARMS-PCR and ddPCR methods were significantly higher than those observed in the non-surgical group, with substantial differences (*P* < 0.001).

### Distribution of genetic alterations observed by the six-gene ddPCR panels

**Fig. 3:**
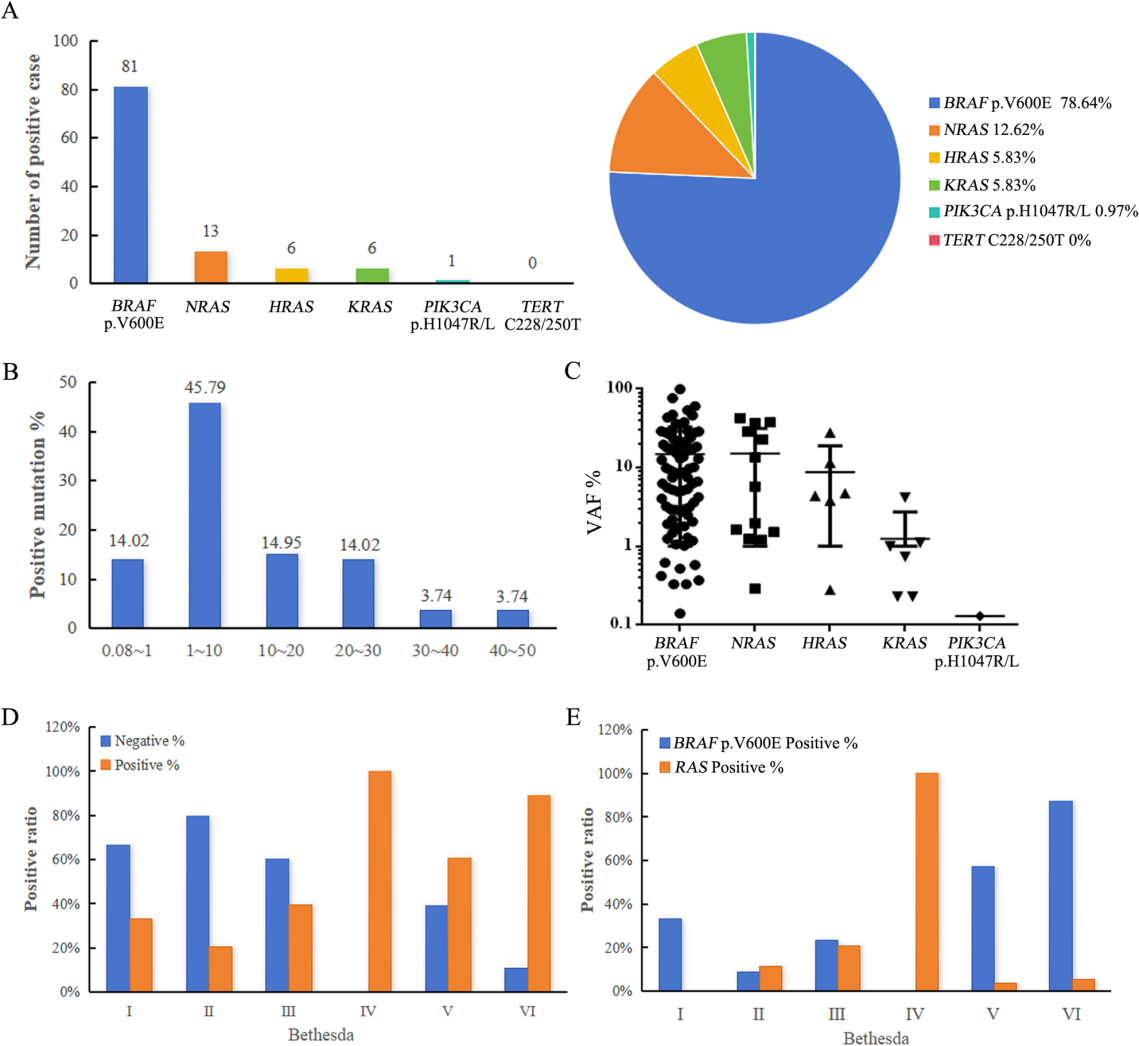
Mutations detected by the six-gene ddPCR assays in the 103 thyroid nodule samples**.** (**A**) This section presents the count of positive cases and their relative frequencies across various mutation classifications identified using six-gene ddPCR assays among thyroid nodule samples. (**B**) It illustrates the distribution of variant allele frequencies (VAFs) for all mutations. (**C)**. This graph depicts VAFs distribution for mutations specific to each gene. (**D)**. This part provides the mutation rate distribution of the six-gene ddPCR assays across different TBSRTC classifications. (**E)**. This panel displays the *BRAF* p.V600E and *RAS* positive rates within varying TBSRTC classifications

A total of 107 mutations were identified across 103 samples with four patients exhibiting two mutations per sample. Of the six genes that could potentially be detected by our ddPCR assays, five were found in the FNA samples: *BRAF* p.V600E (81 of 103; 78.64%), *NRAS* (13 of 103; 12.64%), *HRAS* (6 of 103; 5.83%), *KRAS* (6 of 103; 5.83%), *PIK3CA* p.H1047R/L (1 of 103; 0.97%)(Fig. 3A). The distribution of the obtained VAFs was predominantly within the range of 1–10% (45.79%), with 14.02% of mutations detected at low VAFs (< 1%) (Fig. 3B). Regarding each mutation classification: *BRAF* p.V600E (14.94%±18.80%), *NRAS* (15.08%±16.39%), *HRAS* (8.78%±10.08%), *KRAS* (1.25%±1.48%), and *PIK3CA* p.H1047R/L(0.13%) (Fig. 3C). Gene mutations were more frequently observed in individuals with elevated FNA Bethesda grades. In patients categorized from Bethesda I to VI, the rates of gene mutations were recorded at 33.33%, 20.51%, 39.53%, 100.00%, 60.71% and 89.09%, respectively(Fig. 3D). Interestingly, 3 Bethesda Ⅳ samples showed 100% mutation of *RAS* genes(Fig. 3E).

### ddPCR compared to ARMS-PCR for *BRAF* p.V600E mutation detection

Among the 210 FNA specimens, ddPCR identified *BRAF* p.V600E mutations in 38.57% (81/210) of cases, while the detection rate by ARMS-PCR was 36.19% (76/210).The difference in detection rates between the two techniques was statistically significant (χ2 = 189.69, *P* < 0.001), indicating that the P value specifically reflects the difference in mutation detection performance between ddPCR and ARMS-PCR, rather than differences in clinical subgroup prevalence (Table 3). Among the five ARMS-PCR-negative yet ddPCR-positive specimens, Bethesda grades were distributed as follows: one each in categories Ⅱ, Ⅲ and Ⅴ, and two in category Ⅵ. One of these cases underwent surgery, and the postoperative pathology was papillary thyroid carcinoma. The *BRAF* p.V600E mutation frequency in these 5 missed specimens ranged from 0.37% to 2.96%. Although 3 of them were above 1%, they were still not detected by the ARMS-PCR method (Table 4).

**Table 3:** Compares the effectiveness of ARMS-PCR and ddPCR in detecting *BRAF* p.V600E mutations within FNA specimens

ddPCR BRAF *p*.V600E	ARMS-PCR BRAF *p*.V600E			
	Positive	Negative	Total	*P* < 0.001
Positive	76	5	81	
Negative	0	129	129	
Total	76	134	210	

**Table 4:** The clinical validity of five patients displaying inconsistent results between ARMS-PCR and ddPCR

Patient ID	sex	age	Bethesda	ARMS-PCR	ddPCR	ddPCR ratio%	Surgical pathology
G2023-29539	Female	40	Ⅵ	WT	*BRAF* p.V600E	0.62	PTC
G2022-0271	Male	45	Ⅱ	WT	*BRAF* p.V600E	1.80	/
G2022-0407	Female	37	Ⅴ	WT	*BRAF* p.V600E	0.37	/
G2022-0421	Female	35	Ⅲ	WT	*BRAF* p.V600E	2.96	/
G2022-0431	Male	31	Ⅵ	WT	*BRAF* p.V600E	2.79	/

### Diagnostic value of FNA and molecular testing

With surgical pathology serving as the gold standard, we conducted a comparative analysis of the clinical validity of FNAC, ARMS-PCR, ddPCR (*BRAF* p.V600E), and ddPCR (six gene). Among the 49 surgically excised thyroid nodules, 42 were identified as malignant tumors, comprising 27 classic papillary thyroid carcinomas (PTC), 13 papillary thyroid microcarcinomas (PTMC), and 2 follicular variant papillary thyroid carcinomas (FVPTC); while the remaining seven were benign: 3 nodular goiters, 2 follicular nodular lesions, and 2 Hashimoto’s thyroiditis. The details of the mutations detected and their frequencies for each Bethesda category of FNA nodules were provided in the supplementary table S1.

Among the 42 malignant tumors, the 33 *BRAF* p.V600E–positive tumors(positive rate 78.57%) exhibited a mean largest diameter of 0.79 ± 0.08 cm, whereas the 9 *BRAF* p.V600E wild-type lesions measured 0.98 ± 0.14 cm. The results indicated that FNAC plus ddPCR (six genes) achieved high sensitivity yet low specificity for preoperative differentiation of benign versus malignant thyroid nodules, whereas ARMS-PCR and ddPCR-*BRAF* p.V600E displayed the opposite profile—robust specificity but limited sensitivity, with the differences reaching statistical significance (*P* < 0.05) (Table 5).

**Table 5:** Diagnostic efficiency of FNA, ARMS-PCR, ddPCR and the combination of FNA and ddPCR in distinguishing thyroid malignancies

Examination	Malignant (*n* = 42)	Benign (*n* = 7)	Sensitivity	Specificity	PPV	NPV	Accuracy
FNA Bethesda			**85.71**	**71.43**	94.74	45.45	83.67
II: benign	0	2					
III: AUS/FLUS	5	2					
IV: FN/SFN	1	1					
V: SM	10	2					
VI: maligancy	26	0					
ARMS-PCR-*BRAF* p.V600E			**76.19**	**100**	100	41.18	79.59
Positive	32	0					
Negative	10	7					
ddPCR-*BRAF* p.V600E			**78.57**	**100**	100	43.75	81.63
Positive	33	0					
Negative	9	7					
ddPCR-six gene			**85.71**	**71.43**	94.74	45.45	83.67
Positive	36	2					
Negative	6	5					
ddPCR *BRAF* p.V600E+FNAC			**97.62**	**71.43**	95.35	83.33	93.88
Positive	41	2					
Negative	1	5					
ddPCR-six gene+FNAC			**100**	**71.43**	95.45	100	95.92
Positive	42	2					
Negative	0	5					
ddPCR-six gene for ITNs			**81.25**	**60**	86.67	50	76.19
Positive	13	2					
Negative	3	3					
ddPCR-six gene+FNAC for ITNs							
Positive	16	2	**100**	**60**	88.89	100	90.48
Negative	0	3					

ITNs: indeterminate nodules, icluding Bethesda III/IV/V

A comparative analysis was performed to evaluate the diagnostic performance of ddPCR (*BRAF* p.V600E) plus FNAC versus the six-gene ddPCR panel combined with FNAC for differentiating benign and malignant thyroid nodules. The six-gene ddPCR integrated with FNAC achieved a sensitivity of 100% and an overall accuracy of 95.92%. In cytologically indeterminate nodules (Bethesda III–V), this combined approach maintained a sensitivity of 100% with an accuracy of 90.48%, significantly outperforming ddPCR (*BRAF* p.V600E) alone, the six-gene ddPCR panel alone, or FNAC alone (*P* < 0.05) (Table 5).

In the Bethesda III–V subgroup, the six-gene ddPCR panel alone yielded a sensitivity of 81.25%, specificity of 60.00%, and accuracy of 76.19%. When combined with FNAC, diagnostic performance improved markedly, with sensitivity increasing to 100% and accuracy to 90.48% (*P* < 0.05).

Additionally, we identified six Bethesda V/VI nodules that were negative by the six-gene ddPCR panel in preoperative FNA samples. To clarify whether these were false negatives or truly mutation-negative cases, we performed ddPCR analysis on the corresponding surgical specimens. As detailed in Table 6, three of these six cases were found to harbor *BRAF* p.V600E mutations in tissue DNA (MAFs: 1.96%, 5.2%, and 0.85%). All three cases were confirmed to be papillary thyroid carcinoma (PTC) or its follicular variant (FVPTC), with nodule diameters ranging from 0.6 cm to 1.0 cm. The remaining three cases remained mutation-negative in tissue, including two malignant lesions (PTC and FVPTC) and one benign lesion (nodular goiter). These findings suggest that the ddPCR panel may have missed low-abundance mutations in FNA samples, possibly due to tumor heterogeneity, limited tumor cell content, or sampling variability.

**Table 6:** ddPCR results in postoperative tissue samples and histopathological findings of six cytologically malignant but ddPCR-Negative Cases

*Patient ID*	*Sex*	*Age*	*Surgical sample*				
			ddPCR	ratio%	Pathology	Nodules number	Nodules diameter
G2023-351	Male	34	*BRAF* p.V600E	1.96	PTC	1	0.6 cm
G2022-355	Female	60	*BRAF* p.V600E	5.2	PTC	2	1/0.5 cm
G2022-170	Male	47	WT	/	FVPTC	1	1.3 cm
G2022-059	Female	50	WT	/	PTC	1	1.5 cm
G2022-368	Male	60	WT	/	nodular goiters	/	/
G2022-093	Female	32	*BRAF* p.V600E	0.85	PTMC	1	0.6 cm

## Discussion

This study employed a panel of four multiplex ddPCR assays covering six clinically relevant genes to perform mutational analysis on thyroid nodule samples, including *BRAF* p.V600E, *TERT* C228/250T, *NRAS* (codons 11–16, 59–64), *HRAS* (codons 11–16, 58–64), *KRAS* (codons 9–15, 58–63) and *PIK3CA* p.H1047R/L variants, which are closely associated with thyroid carcinogenesis. Compared with ARMS-PCR, the detection limit of ddPCR was reduced to 0.1%, and 5 cases of *BRAF* p.V600E low abundance mutations (VAF 0.37–2.96%) missed by ARMS-PCR were successfully captured, including 1 case confirmed as PTC by postoperative pathology. This result is consistent with previous reports [28, 29], suggesting that ddPCR can significantly reduce the risk of false negatives in samples with low tumor cell content or high heterogeneity. A significant correlation was observed between Bethesda classification and mutation prevalence, with mutation rates showing a progressive increase across higher Bethesda categories. Although the overall detection rate of *RAS* mutations is only 12.6%,lower than the findings of Zhang et al. [30], *NRAS* codon 61 and *KRAS* codon 12/13 are detected in low-grade follicular lesions, and caution should be exercised about their potential to evolve into FTC or FV-PTC [31, 32]. It is worth noting that three Bethesda IV samples showed 100% *RAS* mutations, and one of them was confirmed to have minimally invasive FTC after surgery, indicating that *RAS* mutations have “targeted diagnostic” value in follicular lesions. Notably, one case showed a positive *BRAF* p.V600E result by ddPCR while being classified as Bethesda II on cytological examination. As histological confirmation was not available, a false-positive ddPCR result cannot be completely excluded. Nevertheless, this discordance may also be explained by the presence of an occult papillary thyroid microcarcinoma, whose neoplastic cells were not captured during fine-needle aspiration. Considering the limited sampling nature of FNA and the potential spatial heterogeneity of small thyroid carcinomas, molecular–cytological discordance is biologically plausible and has been reported in prior studies. These findings highlight the complementary value of molecular testing in identifying clinically occult malignancies that may be missed by cytology alone. No *TERT* promoter mutations were detected in the current series; accordingly, *BRAF* p.V600E*/TERT* or *NRAS/TERT* co-mutations were not observed. Published data from larger cohorts report these co-mutations at frequencies below 1% [33, 34]. This study further confirmed that *BRAF* p.V600E remains the predominant driver mutation in Chinese patients with papillary thyroid carcinoma (PTC), with a prevalence of 78.57%, which is notably higher than the reported frequency of 45–60% in Western populations. This finding is consistent with previous studies and highlights the geographic and ethnic variability in the molecular landscape of PTC.

In our cohort, *BRAF* p.V600E–positive tumors exhibited a numerically smaller mean diameter and were more frequently associated with higher cytological grades, suggesting that this mutation may represent an early oncogenic event in PTC development. However, formal statistical analysis did not demonstrate a significant difference in tumor size between *BRAF* p.V600E-positive and -negative groups. This lack of statistical significance may be partly attributable to the limited sample size, which may have reduced the statistical power to detect subtle differences. Larger prospective studies are therefore warranted to further clarify the relationship between *BRAF* p.V600E mutation status and tumor growth dynamics.

From a diagnostic perspective, ddPCR detection of *BRAF* p.V600E alone achieved a specificity of 100%, although the sensitivity was limited to 78.57%, consistent with previous reports. Importantly, the six-gene ddPCR panel significantly improved diagnostic sensitivity to 85.71%, and when combined with cytological evaluation, the sensitivity further increased to 100%, markedly outperforming the ARMS-PCR combination strategy (76.19%). These findings underscore the substantial diagnostic benefit of integrating cytological and molecular data, supporting the clinical utility of multimodal approaches for improving the diagnostic accuracy of thyroid nodules, particularly in indeterminate cases.

Several limitations of this study should be acknowledged. First, this was a single-center retrospective analysis, and surgical pathology was unavailable for a proportion of cytologically benign nodules, which may have resulted in an overestimation of the negative predictive value. Multicenter studies involving larger and more diverse patient populations are therefore needed to validate the generalizability of our findings. Second, the current ddPCR panel did not include fusion genes such as RET/PTC or PAX8/PPARG, which represent important oncogenic drivers in thyroid cancer. Future integration of RNA-based ddPCR assays could enable combined DNA–RNA detection and further expand diagnostic coverage. Third, long-term follow-up data were limited, precluding an assessment of the association between specific molecular alterations and clinical outcomes or prognosis.

Finally, this study was based on residual DNA samples obtained after routine clinical molecular testing rather than a prospectively collected surgical cohort. Consequently, a substantial proportion of cases, including some Bethesda V and VI nodules, lacked postoperative histopathological confirmation due to referral to other institutions or delays in elective surgery. This limitation restricted comprehensive genotype–phenotype correlation analyses and may have influenced the evaluation of diagnostic performance. Moreover, although the diagnostic utility of the six-gene ddPCR panel was explored in indeterminate thyroid nodules (Bethesda III–V), the number of surgically confirmed cases in this subgroup was limited. Therefore, the calculated sensitivity, specificity, PPV, and NPV should be interpreted with caution. Prospective studies with larger cohorts and complete surgical follow-up are warranted to further validate the clinical utility of this approach.

In conclusion, the six-gene ddPCR technology demonstrates superior sensitivity, specificity, and detection range, particularly excelling in low-frequency mutation analysis. This approach provides a novel paradigm for precision diagnosis in thyroid disease, serving as a highly effective complement to conventional cytology. When interpreted in combination with cytological findings, the six-gene ddPCR platform may provide additional molecular information that supports risk stratification and clinical decision-making in thyroid nodules, while acknowledging that some detected mutations, such as *RAS* alterations, are not specific to malignancy.

## Supplementary Information

Below is the link to the electronic supplementary material.

Supplementary Material 1

Supplementary Material 2

## Data Availability

No datasets were generated or analysed during the current study.
